# Sociotechnical Misalignments in Hospital AI System Implementation: Qualitative Case Study

**DOI:** 10.2196/87534

**Published:** 2026-08-10

**Authors:** Adrian Yeow, Jennifer Cleland, Christina Soh, Candice Balete

**Affiliations:** 1 School of Business, Singapore University of Social Sciences, 463 Clementi Road, Singapore, Singapore, 65 62480221; 2 Lee Kong Chian School of Medicine, Nanyang Technological University, Singapore, Singapore; 3 National Healthcare Group, Singapore, Singapore; 4 Nanyang Business School, Nanyang Technological University, Singapore, Singapore

**Keywords:** artificial intelligence, AI, hospital information systems, public hospitals, hospital personnel, delivery of health care, mobile apps, information dissemination, technology

## Abstract

**Background:**

Implementing AI into real-world health care settings is known to be challenging, particularly regarding how well AI is embedded into the existing knowledge, practices, and procedures of a context. Understanding this process is critical for maximizing the successful implementation of AI tools and planning the time, costs, and resources needed for their successful implementation.

**Objective:**

This study sought to examine the contextual challenges of implementing an AI chatbot, ChatAI (which provided quick access to clinical and operational information without relying on the intranet), in a large tertiary government hospital by analyzing the impact of sociotechnical factors on its sustained use.

**Methods:**

We used an instrumental case study approach, utilizing interviews and meeting minutes. A total of 16 semistructured interviews were conducted with the implementation team and hospital staff who interacted with ChatAI. Interviews were audio-recorded and transcribed. Sociotechnical systems (STS) theory, specifically Davis et al’s (2014) framework, was adopted to examine ChatAI’s implementation and use.

**Results:**

Multiple misalignments among 5 of Davis et al’s sociotechnical elements (goals, people, processes, technology, and infrastructure) limited ChatAI’s user adoption and sustainability. Although the hospital’s innovation center team attempted to address these initial misalignments, contextual changes such as new regulatory mandates, infrastructure changes, and evolving stakeholder practices introduced further misalignments between ChatAI and the hospital—eventually leading to its discontinuation.

**Conclusions:**

This study highlights how sociotechnical misalignments can undermine the use and sustainability of large-scale implementation of AI systems. These findings will inform future efforts to implement AI tools in real-world health care settings, increasing awareness of the need to align sociotechnical dimensions of goals, people, processes, technology, and infrastructure. It highlights the particularly challenging aspect of aligning continually evolving infrastructure with regulatory requirements. Future research should focus on how infrastructure and infrastructure changes, as well as external regulatory requirements, influence AI implementation and use.

## Introduction

There are many complex challenges in implementing AI into real-world health care settings [1-6]. There are technical problems with the tools themselves (eg, poor generalizability, true and false negatives and positives, and reliability). Another, arguably greater, challenge relates to how well an AI system is embedded within the local sociotechnical context of implementation [7-10]. AI tools do not produce benefit on their own; they must be embedded within an ensemble of knowledge, practices, and procedures that govern the use case for the tool, including the conditions under which it is likely to be effective or not. Previous studies have found that the efficacy of AI tools relied on the flow of information among clinicians, trust and perceptions of utility of the tool, and broader workflow processes (eg, Sandhu et al [11]).

One of the major factors leading to the failed uptake of AI systems and tools is an inadequate understanding of sociotechnical aspects, especially how individuals and organizations adopt new technology [12]. Despite increasing numbers of studies focusing on this issue [13-17], there remains a limited understanding of the knowledge, practices, and procedures necessary when implementing AI tools into clinical workplaces. Yet this understanding is critical in terms of maximizing the successful implementation of such tools and planning the time, costs, and resources needed to support successful real-world implementation efforts.

Thus, our objective in this study was to describe and understand the contextual challenges of implementing an AI-enabled chatbot app, ChatAI, in a large tertiary care hospital. ChatAI provided quick access to a central repository of clinical and operational information without relying on the intranet (refer to Methods for further description). We examined the feasibility, penetration, acceptability, and sustainability [18] of ChatAI.

**Figure 1.**
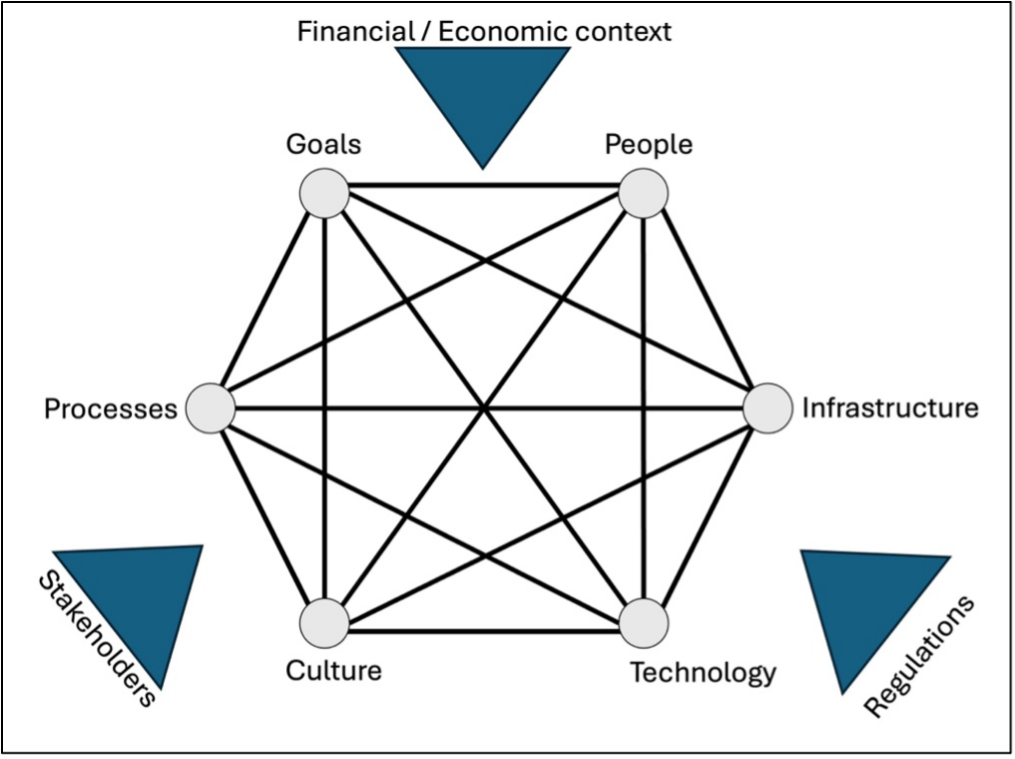
A visual representation of the sociotechnical system model reproduced from Davis et al [20], with permission from Elsevier. "Culture" was retained for framing but was not found to be relevant to the observed misalignments, and “Financial/Economic context” was shortened to “Financial context” for our study.

To aid conceptual generalizability, we used sociotechnical systems (STS) theory as a model against which ChatAI’s implementation into workflows could be better understood. This approach advocates consideration of both technical and social factors when seeking to promote change within an organization, such as the introduction of new technology. It positions organizations as complex systems comprising many interdependent factors. It acknowledges that change in one part of the system will affect, or require changes in, the other aspects of the system [19]. Specifically, we adapted Davis et al’s [20] STS framework (Figure 1) to examine the linkages and relationships between how ChatAI was used and integrated within the hospital’s existing STS. This allowed us to consider our data within the interrelated elements of goals, people, processes, technology, and infrastructure that are embedded within 3 contextual factors.

## Methods

### Study Context and Design

The setting of our study was a large government tertiary hospital with about 2000 beds in a Southeast Asian country. In 2016, the hospital launched the Innovation Center (IC) to improve hospital productivity and patient care. Our case study focuses on one of the health care technological projects launched by the IC: the implementation of an AI chatbot to assist user access to hospital information.

The AI chatbot was created by a startup company, ChatAI (a pseudonym), that provided solutions integrating AI technology with electronic medical records and hospital information systems. ChatAI ran a successful pilot with the IC for their emergency department (ED) physicians to access roster, pharmacy, and guideline information (refer to below for details). After the pilot’s demonstrated success, the IC team proposed to adopt ChatAI across the hospital for both physicians and nurses. This full implementation project began in April 2023 and went live on December 11, 2023. In parallel, the research team was invited to study the impact of ChatAI.

We were invited to conduct our case study in 2023 and began collecting data from March 2024 to May 2025. We adopted an instrumental case study approach, critically examining a real-life setting using multiple sources of data to capture context and extend knowledge on AI implementation in health care settings [21]. This case study follows the COREQ (Consolidated Criteria for Reporting Qualitative Research) to ensure transparency and rigor. See Checklist 1 for the COREQ checklist.

### Data Collection

We collected interview data through semistructured interviews and archival data. Interview participants were selected through purposive sampling to capture a range of perspectives about the ChatAI design and implementation, adoption, and use. Participants included ChatAI users, including physicians and nurses across different ranks and hospital departments, the IC team (who were in charge of the project implementation), and a leadership representative from the ChatAI team. We took care to include active and less active ChatAI users for the interviews. Additionally, a snowball sampling technique [22] was used, whereby suggestions were elicited from interview participants about other eligible individuals who might have relevant insights. The interview guide was semistructured, developed by drawing on the extensive literature on implementing AI into health care practice. It was used in an iterative manner, refined based on preliminary analysis of data from earlier interviews.

Interviews were conducted by researchers (AY and CB) either face-to-face at the hospital or via Zoom (Zoom Video Communications, Inc) and Microsoft Teams between March 2024 and May 2025. They were conducted in English, lasted approximately 30 minutes to an hour, and were audio-recorded with participant consent. Only audio recordings were retained from the platforms. Our source of archival data was the minutes of meetings between the IC and ChatAI teams. We collected minutes from 19 meetings between May 2024 and September 2025. Although the ChatAI system was officially sunset on February 28, 2025, meetings continued after this date to conduct postmortem reviews and stakeholder reflections on the implementation, including exit discussions. These post shutdown meetings formed part of the archival data analyzed in this study.

### Data Analysis

Interview data were transcribed, fully anonymized, and then analyzed in MAXQDA 24 (VERBI GmbH) [23]. Initial analysis was inductive and thematic [24]. Researcher AY conducted the first round of coding. CB then coded several transcripts independently. Working together and in discussion with the wider team, they developed a coding framework to be used to code all data. Analysis progressed via regular team (online) meetings, in which ongoing coding and comparisons were explored. During this process, we were struck by the social and technical aspects of the process. We therefore extended beyond simple thematic analysis to a more deductive approach and coded the data using Davis et al’s sociotechnical theory [20] (Multimedia Appendix 1, Coding Tree—STS concepts), including identifying contextual triggers and misalignments. Analysis was also guided by the chronology of the implementation process to trace the complex interactions across different elements within the system and its wider context over time. We relied on the meeting minutes to triangulate and validate the chronology of the implementation process and interactions. The interpretation and analysis of themes were shared and discussed at full team meetings, with any divergences discussed and revisited in detail until consensus was achieved. This resulted in our final coding tree (Multimedia Appendix 1), with illustrative quotes for the codes presented in Multimedia Appendix 2. We engaged in synthesized member checking by sending an outline of our interpretations to the IC team, inviting them to assess whether the findings reflected their experiences and perspectives, and to provide any additional feedback.

### Ethical Considerations

This study adhered to ethical standards for research involving human participants. Ethics approval for this study was obtained on February 21, 2024, by the National Healthcare Group, Domain Specific Review Board, Singapore (reference number 2023/00410).

All participants provided informed consent prior to their participation in the study by signing an informed consent form. Participation in the study was voluntary. It was made clear to participants that they could withdraw from the study without impacting their standard routine work, training, or career assessment.

To ensure privacy and confidentiality, all collected data were anonymized and deidentified prior to analysis. Any personally identifiable information was removed or coded to protect participant identities, and access to interview recordings and transcripts was limited to authorized members of the research team. No images included in the manuscript or supplementary materials allow the identification of individual participants. No compensation was provided for participation in the study.

## Results

We conducted 16 interviews from March 2024 to May 2025, and the summary of the interviews conducted can be seen in Table 1.

**Table 1.** Summary of interview sessions with clinical users, the IC (Innovation Center) team, and the ChatAI team conducted at a tertiary care hospital (2024-2025).

Interview group	Interviews conducted, n (%)	Interviewees, n (%)
IC team	3 (18.8)	3 (18.8)
ChatAI team	1 (6.3)	1 (6.3)
Junior physicians (ChatAI users)	5 (31.3)	5 (31.3)
Senior physicians (ChatAI users)	2 (12.5)	2 (12.5)
Nurses (ChatAI users)	3 (18.8)	3 (18.8)
Pharmacists (ChatAI users)	2 (12.5)	2 (12.5)
Total	16 (100)	16 (100)

### Pilot Project and the ChatAI’s Chatbot System Overview

In early 2020, the IC clinical lead contacted ChatAI to run a pilot project in the hospital’s ED. The goal was to demonstrate how the ED could benefit from ChatAI’s chatbot system that enabled quick access to the hospital’s central repository of clinical and operational information without relying on the intranet. The ED pilot project began in May 2020 and ended in mid-2021. It was acceptable to users and demonstrated measurable utility (eg, finding roster information was cut down from 2‐3 min to 5-10 s). Overall, the ChatAI ED pilot project was perceived as a success, and that positive outcome led the way for the eventual rollout of ChatAI across the hospital.

It is useful to give some technical details here. The chatbot system is a mobile app that uses a natural language processing–enabled chat interface to retrieve information from existing databases and other internet sources. The chatbot system’s potential users include those who require access to operational and clinical guideline data via a chat interface on their mobile phone apps. The chatbot system is trained on the hospital’s content and can provide access to the following information: hospital directory, hospital rosters, drug information, pharmacy information (formulary), laboratory test catalog, and hospital guidelines and protocols. It has various customized functionalities, such as a system-wide broadcast and integration with other online information providers (eg, UpToDate). Refer to Multimedia Appendix 3 for the chatbot system architecture.

### Hospital-Wide Chatbot Implementation (2023-2025)

#### Salient Contextual Factors

##### Stakeholders

For ChatAI’s hospital implementation, the IC clinical lead and the IC team were able to secure management’s approval for the project. With management approval, the IC team could then engage with the external health care IT agency (“HITA”), which managed the hospital’s IT system and had to vet all new IT systems.

##### Financial Context and Regulations

Despite the pilot’s positive outcomes, there was concern about whether ChatAI would improve work efficiency for the entire hospital. Thus, the management team decided to adopt a financially conservative approach and buy only 1000 licenses in the first year, which could rise to 2000 licenses in the second year depending on use. This meant that at any time, only some of the 6000 staff would be given access to ChatAI (eg, senior physicians were originally prioritized for ChatAI licenses, while residents did not have access). Another key financial consideration was the cost of the infrastructure setup. Due to the government’s data security regulation governing hospital databases, the IC team had to install a version of ChatAI on the secure cloud system (M-Cloud) so that it could access hospital data. ChatAI’s installation on M-Cloud meant significant setup and ongoing hosting costs.

### Initial Misalignments

#### Goals Versus People and Technology

The IC team’s goal for the ChatAI project was to assist users in accessing information efficiently. This goal was also the focus of other parallel activities within the hospital. Specifically, since the time of the ChatAI pilot, the hospital had implemented new systems by which users could access information (eg, the introduction of a new electronic medical record system and a new shared folder system). The IC team had no power to convince users and departments across the hospital to buy into the ChatAI system, and with only 1000 licenses, only selected users had access anyway.

#### New Technology Versus People and Processes

The functionality of ChatAI depended on the data it held. Data upload and updating depended on department representatives (operational executives or clinicians) who were responsible for identifying, uploading, and updating department content in ChatAI. This meant additional data-related work for the department representatives on top of their existing workload, with no obvious direct benefit to them for doing so.

#### New Technology Versus Infrastructure and People

The hospital had a new enterprise shared folder system that enabled users to access shared content from other departments. Some of this shared content had to be converted for data protection. For example, the pharmacy department had converted their pharmacy data from Excel to PDF format. However, the ChatAI system could not parse the data from PDF files to provide direct responses—instead, it could only reply to the information request with a PDF attachment. This was a new misalignment between ChatAI and the hospital’s data infrastructure. It also resulted in misalignment between ChatAI and users as they had to take extra steps to access the information they required.

#### Outcomes

These misalignments between ChatAI and other sociotechnical dimensions led to low adoption and use among those selected to use the system (Multimedia Appendix 4).

### IC Team’s Effort to Address Misalignments

The IC team originally only issued ChatAI licenses to selected, senior physicians across departments. However, many of these physicians considered ChatAI redundant as they always had access to required information via their office computers. The IC team then switched the user strategy to focus on residents and other users who were constantly on the move around the hospital and needed a mobile means of accessing information (aligning goals vs people and technology). Thus, they began issuing ChatAI licenses to residents, house officers, and nurses. They also complemented this by increasing the awareness of ChatAI among these users (eg, conducting training workshops, sending marketing emails, and putting up posters).

The IC team also aligned ChatAI with these users by ensuring that relevant data were made available on ChatAI (eg, they worked with the residency program head to upload the residency handbook and processes—information that residents would need as part of their work). Finally, the IC team replaced the inactive ChatAI users with new active ones—they deactivated inactive licenses and issued them to the new target users. Overall, the IC team’s efforts at aligning new technology vs people and processes were relatively successful, as evidenced by the 4-fold increases in license activations and queries (Multimedia Appendix 4).

### Contextual Triggers Creating First- and Second-Order Misalignments

While the IC team attempted to address the initial misalignments, contextual conditions related to regulations and stakeholders triggered a series of misalignments (Multimedia Appendix 5). We trace how these misalignments led to negative outcomes for the hospital’s ChatAI use.

#### Regulations Triggering New Technology Versus People and Processes Misalignment

After the ChatAI implementation was completed, HITA informed the IC team that all subsequent hospital data uploaded to ChatAI had to be vetted and approved by a HITA representative. HITA explained that this ChatAI data approval process was part of the hospital’s data control regulation. Thus, all department representatives had to submit a request form for any data upload to ChatAI. This form would be routed to HITA, and they could only upload the data to ChatAI upon HITA’s approval. This created additional work for the department representatives in charge of department data and further reduced their willingness to update ChatAI. Second, it led to delays in ChatAI data updates as HITA’s vetting and approval process could not keep up with the volume of requests.

#### Regulations Triggering New Technology Versus Infrastructure Misalignment

When ChatAI was implemented, the hospital’s roster data was managed by a legacy system. ChatAI was integrated with that legacy system to provide up-to-date roster schedules. Roster data were consistently the most frequently accessed content by ChatAI users. However, the hospital’s IT team planned to transition from the legacy system to a new roster app around July 2024. The IC and ChatAI teams reached out to the hospital IT team to ensure that ChatAI was integrated with the new roster app. Unfortunately, this was not possible for technical and regulatory reasons. Technically, the new roster app did not have an integration tool. In terms of regulations, the roster app could not interface with ChatAI as it was not on a government “whitelisted” web domain. As a result, ChatAI lost access to the hospital’s updated roster data after the hospital transitioned to the new roster app.

#### Stakeholders Triggering New Technology Versus Processes Misalignment

With the implementation of the hospital’s mandatory shared folder system, some departments began to adopt some of its advanced functions (eg, dynamic folders) to share content with their team members. This shared folder system was part of a larger enterprise communication platform, and team members could also attach files from the folders when contacting each other. This was also rolled out as a mobile enterprise app that all team members could access. As these departments adopted the shared folder system (on the mobile app) as part of their data-sharing processes, ChatAI became redundant.

#### Second-Order Misalignments

The first-order misalignments described above led to reduced quality of ChatAI data (specifically timeliness and completeness) because data were either not uploaded or updates took more time. In addition, with the loss of roster data, many active ChatAI users were unable to contact the correct persons using ChatAI’s data and they switched to the new roster app. Similarly, those departments that switched to the shared folder system discontinued their use of ChatAI. These were the second-order misalignments between new technology vs people and processes (Multimedia Appendix 5).

### Ending the ChatAI Project

At the end of the first year of operations, hospital management had to make the decision whether they should continue the ChatAI project and increase the subscription to 2000 licenses. Archival meeting records indicated that the IC team conducted a cost-benefit analysis of the ChatAI project to inform this decision. In terms of benefit, the IC team found that the ChatAI system was significantly underused. In terms of cost, the hospital was not only paying for the ChatAI licenses but also the (substantial) monthly M-Cloud hosting fees and the manpower to support the ChatAI users. In addition, the IC team realized that ChatAI’s functions could be (indeed were already being) replaced in part by other hospital systems. The IC team recommended sunsetting the ChatAI system to the hospital management and transitioning all ChatAI users to existing systems. The hospital management accepted the IC team’s recommendation, and the ChatAI system was shut down on February 28, 2025.

## Discussion

### Principal Findings

We sought to examine the contextual challenges of implementing an AI chatbot, ChatAI, in a large tertiary government hospital, using longitudinal analysis through a STS lens. We found that misaligned sociotechnical dimensions of the large-scale implementation had unintended consequences for ChatAI’s use and sustainability, leading to its decreasing use and eventual shutdown. Some of the misalignments arose from “good-intentioned” internal decisions, such as a conservative phased approach to implementation, which limited the number of ChatAI users and thereby translated into limited use and limited engagement. Other misalignments were triggered by external regulatory requirements such as the need for all data to be first vetted by the regulatory body before being uploaded to the AI app. While the implementation team was able to address some of the misalignments arising from internal decisions, they were not able to effectively mitigate the misalignments arising from the organization’s evolving infrastructure and external regulatory triggers. Our study of the failed sustainability of an AI-enabled chatbot system provides a cautionary tale of how successful small-scale pilots may not translate into successful large-scale implementations.

### Comparison to Prior Work

Current literature on sociotechnical studies of AI system implementations has shown that the alignment of the AI tool with workflows and other aspects of the work environment to be a critical factor in implementation success [8,10]. Others have discussed the need to navigate between the social and technical elements within an organization during implementation [9]. While our results validated these insights, we show that it is not sufficient to focus only on the alignment of sociotechnical dimensions within the organization but that it is also critical to take into account the impact of the wider context interactions on these internal dimensions. Specifically, our findings highlight that the dimension of infrastructure (which is evolving) and the contextual factor of regulations warrant closer attention in the case of AI implementation. Specifically, the AI system’s interoperability and integration with existing and planned infrastructure, as well as regulatory requirements, need to be clarified during implementation planning. In addition, our study highlights the particular role of data embedded in the AI system and the larger infrastructure. AI requires access to updated data to provide the hoped-for benefits. Such data are usually part of the organization-wide infrastructure and are closely regulated in the health care context. The study shows how process difficulties in uploading data to the enterprise database and regulatory hurdles in accessing data negatively affected people and processes, leading to low use.

### Limitations

First, our study was carried out in the context of one hospital in one country, so we cannot assume our findings are fully generalizable to other contexts. However, the case study approach allowed us to illuminate contextually located processes, practices, and experiences that may otherwise be taken for granted and/or remain unexplored, and the main conclusion from the study is pertinent to all health care organizations working to adopt new technology into their systems and structures. Second, and related to this, our use of sociotechnical theory aids transferability, or conceptual generalizability [25], but it will, of course, have foregrounded certain aspects of the data: another lens may have emphasized different aspects of the problem, such as the nature of the power relationships and social dynamics between managers and clinical staff. Third, and related to this, our data collection was guided by the concept of “information power” [26]: we had a focused research aim, recruited participants with direct experience of the ChatAI system, and generated rich data through interviews and archival materials. These factors supported the adequacy of our sample. Fourth, Davis et al’s [20] STS framework was very useful in teasing out different aspects of the data. However, we found that the culture element was not particularly relevant regarding misalignments. While the data did not provide direct insight into why that might be the case, we tentatively suggest it might be due to a shared work culture throughout the hospital. To explore this further, it would be interesting in future studies to compare the adoption of AI across 2 or more different health care institutions using the same theoretical lens, to see if the impact of culture is then apparent. Fifth, we used a combination of interviews and meeting minutes (a form of documentary evidence) to collect empirical data. This is a common triangulation of data sources in management science and education. However, all qualitative data collection approaches have strengths and weaknesses [27]. For example, interviewees may have wished to give socially desirable responses, although the tone and content of the interviews did not suggest this was the case. Finally, our study design had the advantage of allowing us to capture longitudinal processes (eg, how things changed over time) and how the issues changed over time and were influenced by other activities happening in parallel in the hospital.

### Future Directions

Given the fast-moving nature of technology, future research should focus on how changes in existing infrastructure impact focal AI implementation and vice versa and on how AI implementation changes structures and systems. AI regulation is also evolving, and future research should examine how regulatory guidelines, frameworks, and rules translate into practice and influence AI implementation and use in health care. Practically, our findings suggest that it would be good organizational practice to forecast upcoming changes to an organization’s technology portfolio, so that AI implementation can take future infrastructure integration requirements into account. Further, given the relative newness of AI and its regulation in health care, it is likely that there will be some degree of regulatory ambiguity. Hence, engagement with regulatory authorities regarding clarification and compliance with regulatory requirements should begin early and continue through implementation and into operational use.

### Conclusions

Our study examined the contextual challenges of implementing an AI chatbot in a large tertiary government hospital through an instrumental case study. Our qualitative data analysis revealed how sociotechnical misalignments due to contextual changes can undermine the use and sustainability of large-scale implementation of AI systems. These findings inform future efforts to implement AI tools in health care settings by highlighting the challenge of aligning with continually evolving infrastructure and regulatory requirements.

## Supplementary material

10.2196/87534 Multimedia Appendix 1 Coding tree.

10.2196/87534 Multimedia Appendix 2 Illustrative quotes for codes.

10.2196/87534 Multimedia Appendix 3 ChatAI chatbot system architecture.

10.2196/87534 Multimedia Appendix 4 Sociotechnical alignment changes from pilot to after the implementation (green lines are alignment and red lines are misalignments).

10.2196/87534 Multimedia Appendix 5 Sociotechnical first-order and second-order misalignments due to contextual triggers.

10.2196/87534 Checklist 1 COREQ checklist.

## Abbreviations

COREQ: Consolidated Criteria for Reporting Qualitative Research
ED: emergency department
IC: Innovation Center
STS: sociotechnical systems
