# Supplementary material for: Sociotechnical Misalignments in Hospital AI System Implementation: Qualitative Case Study
Source: J Med Internet Res. 2026 Aug 10;28:e87534. doi: 10.2196/87534 (PMC13455577; doi:10.2196/87534)
Supplement: Supplementary file 2 [file jmir-v28-e87534-s002.docx]

**Appendix 2: Illustrative quotes for codes**

| Concepts | Example of quotes from interview |
| --- | --- |
| Financial context and regulations | “But we still had to put everything into the healthcare commercial cloud, which then generated half a million dollars’ worth of ops costs for that secure server. So, on top of that, because in healthcare commercial cloud, because it also runs clinical data and other sensitive data, the running cost is much higher, and the network speed is also much lower.” (IC Clinical Lead) |
| Initial Misalignments | |
| Goals vs. People and Technology | “I used to actually like ChatAI because it was easy to find information. But this was in the era where we didn’t have the Electronic Medical Records System, you know, now the Electronic Medical Records System has really made a big difference... nowadays we have (EMR), and (hospital) Hub and Sharepoint. These two things didn’t exist in 2020 during the prototyping phase.” (Emergency Department Consultant) |
| New Technology vs. People and Processes | “I can't change the content directly from the AI chatbot dashboard module account. Instead, if I want to update a new document, I have to fill in the form and provide the details and the excel link. For the share point updates, it only works if we don't change the file name, then it is ok. But usually, we add the version to the filename - this helps the colleagues who are referring to the document in the share drive to know if the information is the latest. But for ChatAI, this is considered a new document - so for that I have to go to the form to change and request ChatAI to ingest this new document. … these updates occur at different times - so the work of updating can be quite frequent and troublesome to go through the steps.” (Emergency Department Associate Consultant) |
| New Technology vs. Infrastructure and People | “The bot can only read things it can read. So, (if) the data is not structured in a format that can easily read or standardize there is almost no way that we can actually give back a direct reply. …So, we … told them from the very beginning, if they want direct replies, the data needs to be structured in a way that the bot can easily access and read and then update. But a lot of their departments didn't really want to conform to that. So, they're like no, I want to have my data structured this way on SharePoint. I refuse to give you guys a separate thing, because I don't want to maintain it at all. So then, as a result, we wouldn't be able to display it in what we would have preferred, but it doesn't mean that you don't want the information in there, right? So, part of what we're trying to do is make sure that we can still give them a version of it. It may not be a direct reply, but it would at least still be there.” (ChatAI Leadership Representative) |
|  | “Presently, drug use guidelines, clinical practice guidelines, altering medications – these information flow to clinicians – any search will give a long pdf, 15–30-page pdf.” (Pharmacist) |
| Outcomes | |
| IC Team’s efforts to address misalignments | “We went in to do engagement plans, consultations, even going to do road shows, just to get people on board. So it was a lot of effort being spent. We did see a rise in our utility, but it soon also died down as well, because there's no continuation to it.” (IC Project Lead) |
|  | “The rest eventually picked up because we do very drastic, very, very drastic engagement, so trying to get them on board by putting in more information that's relevant. So we do the engagement mainly after we deploy it.” (IC Project Lead) |
| Contextual triggers creating first- and second-order misalignments | |
| Regulations triggering New Technology vs. People and Processes misalignment | “Yeah, I think the big challenge has been that HITA is a giant organization with tons of bureaucracy and very, very layered. So, while ChatAI is a startup and we can move very quickly and maybe, …(the hospital) …is still quite amenable. You don't control HITA. And unfortunately, HITA is the gatekeeper to all of the government's …hospital cloud infra. Yep. And a lot of things that make sense to them don't make sense to us, right? And the way that they do things is a very traditional way. Like you got to file a ticket. And then the turnaround time is back and forth and tons of emails, you know, that kind of stuff.” (ChatAI Leadership Representative) |
|  | “So actually, in the pilot, I think it allows users to just upload from the AI chatbot dashboard module admin, but now we are not allowed. We can only submit the URL to vendor and HITA for them to process. This actually takes about one week in average. So it takes time, and, yeah, it make the entire application not a platform that can be maintained conveniently.” (IC Analyst) |
| Regulations triggering New Technology vs. Infrastructure misalignment | “The government application vendor has not been friendly about extending API – difficult to get API in, and they have been too busy to get API in to set up security for data to flow in.” (IC Clinical Lead) |
| Stakeholders triggering New Technology vs. Processes misalignment | “So, the issue is with the advent of the Electronic Medical Records System, there have been a lot of all these needs that are being met on a single platform because they're all integrated. So, you have secure chat, you have MS Teams chat, everything that's inside, directory-wise which you can easily access on the Electronic Medical Records System. Your intranet is also quite well customized, can get quick access to information. In fact, all the clinical information mentioned to you the local ones, such as the local guidelines, as well as the antibiotic, they are also accessible via our Teams chat now, because our Teams chat is also being used for our day-to-day work, communication and there's Teams folders. So, our department has put relevant clinical guidelines in the Teams folders that we can easily reference already. So basically, where I'm pointing towards is that ChatAI becomes a duplicated service that doesn't offer anything that I don't already have access to. In my clinical practice when I'm working when I need all these things at hand, I'm always one meter away from a desktop or CoW (Computer on Wheels). So, there is no issue with accessibility to IT hardware. Therefore, my use for ChatAI is really less. It doesn't offer me anything that is unique and that cannot be offered elsewhere.” (Emergency Department Consultant) |
| Second-order misalignments | “What is good is that ChatAI came in, everything is in phone, can find on-call roster, can find the doctor’s name itself. … Actually, enjoyed ChatAI in the initial first few (months), just star favorite. … Now, rarely use (ChatAI) because I need to find doctor’s number fast, but now I can’t find it easily.” (General Medicine Resident Nurse) |
