# Supplementary material for: Sociotechnical Misalignments in Hospital AI System Implementation: Qualitative Case Study
Source: J Med Internet Res. 2026 Aug 10;28:e87534. doi: 10.2196/87534 (PMC13455577; doi:10.2196/87534)
Supplement: Supplementary file 3 [file jmir-v28-e87534-s003.docx]

**Appendix 3: ChatAI chatbot system architecture**

The ChatAI system uses a multi-layered architecture – as it is proprietary information, we only have a basic system schematic of the architecture.

1. User interface: This is the front-end interface where users interact with the chatbot – ChatAI has a mobile application that serves as the user interface. This interface enables the users to send their messages, to display chatbot responses, and provide multimedia/interactive links or forms.
2. Message processing layer: This layer enables text cleaning and formatting, language detection, session management (tracking conversation history), and routing rules.
3. NLP engine: The AI layer of the system uses a rule-based, advanced NLP system to interpret conversational input and parse the knowledge database to produce responses. This layer will interpret user messages to determine intent and entities.
4. Knowledge database: This layer contains the knowledge sources used to answer queries. It uses search indexes and LLM-based tools to parse and extract relevant information from the knowledge databases.
5. Content Ingestion: This layer manages the extraction of external data and formats it for the knowledge database.

Figure of ChatAI chatbot system architecture
