# Supplementary material for: Sociotechnical Misalignments in Hospital AI System Implementation: Qualitative Case Study
Source: J Med Internet Res. 2026 Aug 10;28:e87534. doi: 10.2196/87534 (PMC13455577; doi:10.2196/87534)
Supplement: Supplementary file 4 [file jmir-v28-e87534-s004.docx]

**Appendix 4: Socio-technical alignment changes from pilot to post-implementation (green lines are alignment and red lines are misalignments)**

|  |  |  |
| --- | --- | --- |
| Pilot phase alignments (Panel A) | Initial misalignments (Panel B) | Alignments after IC team’s actions (Panel C) |
