# Supplementary material for: Sociotechnical Misalignments in Hospital AI System Implementation: Qualitative Case Study
Source: J Med Internet Res. 2026 Aug 10;28:e87534. doi: 10.2196/87534 (PMC13455577; doi:10.2196/87534)
Supplement: Supplementary file 5 [file jmir-v28-e87534-s005.docx]

**Appendix 5: Socio-technical first-order and second-order misalignments due to contextual triggers**

|  |  |
| --- | --- |
| Contextual Triggers and First-order misalignment (Panel A) | Second-order misalignment (Panel B) |
