# Supplementary material for: Sociotechnical Misalignments in Hospital AI System Implementation: Qualitative Case Study
Source: J Med Internet Res. 2026 Aug 10;28:e87534. doi: 10.2196/87534 (PMC13455577; doi:10.2196/87534)
Supplement: Supplementary file 6 [file jmir-v28-e87534-s006.docx]

**Appendix**

**Appendix 1: COREQ Checklist**

Domain 1: Research team and reflexivity

1. One author/researcher and a research associate conducted the interviews.
2. This author/researcher has a PhD in Business/Information Systems, and the research associate has a Master’s in linguistics.
3. This author/researcher is an associate professor and a faculty in a business school and the research associate was employed as part of the research team.
4. This author/researcher is male while the research associate is female.
5. The author/researcher is trained in qualitative research methods while the research associate is trained in interview data collection and analysis.
6. This author/researcher and the research associate had no prior relationship with the interviewees.
7. The two other authors/researchers are both female. One has a PhD in psychology and is trained in qualitative, quantitative and mixed methods research. The other has a PhD in Business/Information Systems and is trained in qualitative methods research.
8. The participants/interviewees had no prior knowledge of the project.
9. The author/researchers had no specific bias or assumptions of the research topic. The author/researcher was invited to study the project by the project sponsors but had no vested interest in the project.

Domain 2: Study design

10. Framework: We adopted an instrumental case study approach, critically examining a real-life setting using multiple sources of data to capture context and extend knowledge on the AI implementation in healthcare settings.

Participant selection:

11. Sampling: Interviewees were selected based on purposive sampling. We interviewed ChatAI users, including doctors and nurses across different ranks and hospital departments, the IC team (who were in charge of the project implementation), and a leadership representative from the ChatAI team. ChatAI users (both active and less active) provided insights to how and why they used ChatAI. Perspectives of the IC and ChatAI teams provided insight into the AI chatbot design and implementation process. Additionally, a snowball sampling technique was adopted.
12. Method of approach: We approached the interviewees via email – the IC manager and director endorsed our email invitation prior to disseminating the invitation to the interviewees.
13. Sample size and non-participation: We conducted 16 interviews for this study.
14. Non-participation: There were no dropouts.

Setting

15. Setting: We conducted our interviews via online (Zoom or Teams) and in-person at their office.
16. Presence of non-participants: Nobody else was present except for the interviewee and the researchers.
17. Description of sample: The key characteristic of the sample was their role in implementation process of the AI tool on site: from development, to implementation, to end-user.

Data collection

18. Interview guide: We developed a basic interview guide with sets of interview questions for different groups (e.g., IC implementation team, ChatAI users, ChatAI team members). While we did not formally pilot the interview guide, questions were refined over time, based on the content and discussions in earlier interviews. We allowed the interviewees to guide the interview depending on their responses.
19. Repeat interviews: No repeat interviews were conducted.
20. Audio recording: We recorded the interviews (with consent). We did not collect any video recordings.
21. Field notes: We made field notes during and after the interviews.
22. Duration: Most of our interviews were about 30 minutes to an hour in duration.
23. Data Saturation: Data saturation was not used as the criterion for determining sample size. Instead, sampling was guided by the concept of information power. We recruited all eligible participants with relevant experience of the ChatAI implementation across the study site.
24. Transcripts: We did not return our transcripts for comment or correction.

Domain 3: Analysis and findings

25. Number of data coders: There were two data coders.
26. Description of coding tree: Initial analysis was inductive and thematic but as analysis progressed, we were struck by the social and technical aspects of the process. We therefore extended beyond simple thematic analysis, to a more deductive approach and code the data using Davis et al.’s (2014) socio-technical framework including identifying salient contextual factors, contextual triggers, and misalignments. See Appendix 2 for the coding tree.
27. Themes: We focused on identifying salient contextual factors, contextual triggers, types of misalignments, and efforts to address the misalignments.
28. Software: We used MaxQDA (version 2024) to manage our data and data analysis.
29. Participant checking: We presented our narrative findings to the key stakeholders from the hospital’s IC for validation.
30. Quotations: We presented interviewee quotations to illustrate the themes and codes (see appendix 3). Each quotation was identified by the title of the interviewee.
31. Data and findings: There was consistency in our data and findings.
32. Clarity of major themes: The major themes of misalignments, alignment efforts, and triggers were presented in the findings.
33. Clarity of minor themes: There were no descriptions of diverse/minor cases or themes.
