# Supplementary material for: Sociotechnical Misalignments in Hospital AI System Implementation: Qualitative Case Study
Source: J Med Internet Res. 2026 Aug 10;28:e87534. doi: 10.2196/87534 (PMC13455577; doi:10.2196/87534)
Supplement: Supplementary file 7 [file jmir-v28-e87534-s001.docx]

**Appendix 1: Coding Tree**
